# Poverty-Related Adversity and Emotion Regulation Predict Internalizing Behavior Problems among Low-Income Children Ages 8–11

**DOI:** 10.3390/bs7010002

**Published:** 2016-12-29

**Authors:** C. Cybele Raver, Amanda L. Roy, Emily Pressler, Alexandra M. Ursache, Dana Charles McCoy

**Affiliations:** 1 The Institute of Human Development and Social Change, New York University, New York, NY 10012, USA; epressler@gmail.com; 2 Department of Psychology, University of Illinois at Chicago, Chicago, IL 60607, USA; amanda.roy@uic.edu; 3 Department of Psychiatry, Columbia University, New York, NY 10027, USA; amu2116@cumc.columbia.edu; 4 Graduate School of Education, Harvard University, Cambridge, MA 02138, USA; dana_mccoy@gse.harvard.edu

**Keywords:** poverty, attention bias, emotion regulation

## Abstract

The current study examines the additive and joint roles of chronic poverty-related adversity and three candidate neurocognitive processes of emotion regulation (ER)—including: (i) attention bias to threat (ABT); (ii) accuracy of facial emotion appraisal (FEA); and (iii) negative affect (NA)—for low-income, ethnic minority children’s internalizing problems (*N* = 338). Children were enrolled in the current study from publicly funded preschools, with poverty-related adversity assessed at multiple time points from early to middle childhood. Field-based administration of neurocognitively-informed assessments of ABT, FEA and NA as well as parental report of internalizing symptoms were collected when children were ages 8–11, 6 years after baseline. Results suggest that chronic exposure to poverty-related adversity from early to middle childhood predicted higher levels of internalizing symptomatology when children are ages 8–11, even after controlling for initial poverty status and early internalizing symptoms in preschool. Moreover, each of the 3 hypothesized components of ER played an independent and statistically significant role in predicting children’s parent-reported internalizing symptoms at the 6-year follow-up, even after controlling for early and chronic poverty-related adversity.

## 1. Introduction

Internalizing problems of anxiety, depressive symptoms, and withdrawal represent a major obstacle for children’s successful navigation of social and academic demands in their neighborhoods and schools [1]. Epidemiological studies suggest that low-income, ethnic minority children are at higher risk of elevated levels of internalizing problems than are their more advantaged counterparts [2,3,4]. 

Recently, an innovative and rapidly emerging body of evidence across fields of developmental, clinical, and neuroscientific research points to compromises in multiple components of children’s emotional regulation (ER) as key predictors of their risk of internalizing problems [5,6,7]. In the following paper, we draw together methods and findings from these emerging literatures to consider three domains of emotional regulation that reflect an integrated, theoretically-driven definition of ER. Specifically, we draw on recent empirical frameworks suggesting that ER incorporates human attentional orienting to and appraisal of an emotionally evocative stimulus, along with the modulation of accompanying affective response through higher-order executive or cognitive control [8,9]. We capitalize on neuroscientific methods usually deployed in laboratory settings to directly assess low-income children’s ER. Drawing from these recent advances in affective neuroscience, we examine whether three candidate neurocognitive mechanisms of emotion regulation (ER)—including: (i) attention bias to threat (ABT); (ii) accuracy of facial emotion appraisal (FEA); and (iii) subjective negative affect (NA)—appear to be disrupted in the context of poverty. We investigate whether they are individually, and additively, significantly predictive of internalizing problems among a clinically at-risk sample of low-income, ethnic minority children, ages 8 to 11 years (*N* = 338).

What prior research serves to inform our choice of these three ER processes? Our focus on children’s ABT is based on the theory that humans are clearly evolutionarily “wired” to orient and attend to stimuli that have an emotional valence, with extensive laboratory studies demonstrating that adults and children tend to orient to threatening stimuli more quickly than to non-threatening stimuli within a given visual array [10,11]. To measure those attention processes at neurobiological as well as behavioral levels, neuroscientists have pioneered the use of tasks such as the dot probe paradigm, where individuals are asked to identify the location of a target (an asterisk or “dot”) on a computer screen across a large number of trials. Across those trials, pairs of emotionally neutral versus emotionally negative visual images (such as pictures of a kitchen toaster versus of a ferocious, snarling dog) are first presented for very brief periods of time, allowing investigators to compare participants’ response latencies when the location of the dot is congruent versus incongruent with that of emotionally negative stimuli (see the Measures section below, for greater detail). As reflected through the use of the dot probe paradigm in those studies, emotional information clearly not only captures, but also holds individuals’ attention, making it harder for them to disengage from emotionally valenced stimuli than non-emotional stimuli. Higher ABT using the dot probe paradigm is associated with difficulty modulating anxiety and fear, as illustrated by a growing literature on attention to threat cues among individuals struggling with mood disorders, e.g., high anxiety [12,13]. Based on this emerging literature, we expect that children’s attention bias to emotionally negative and threatening cues using the dot probe paradigm is likely predictive of significantly higher risk for internalizing behavioral difficulty [14,15]. This process may be heightened for children living in households struggling with chronic poverty; as such, children’s increased risk of exposure to adversity (such as greater levels of community and family violence and instability) may exacerbate (or moderate) the ways that biased attention towards threat may increase their risk for psychopathology [16,17].

As children transition from early through middle childhood, they develop increasing skill in not only orienting to emotional stimuli but also in identifying and labeling others’ emotions [18]. Individual differences in FEA are predictive of grave social and clinical outcomes, including higher risk of rejection from peers, feelings of failure in social situations, social isolation, and loneliness [6,7,19]. In addition, lower levels of accuracy in identifying emotions by using appropriate emotion labels has been found to be robustly predictive of higher risk of internalizing difficulty among younger children [20,21]. We expect to find similar relations between higher accuracy in emotion appraisal and lower levels of parent-reported internalizing problems for our sample of low-income, urban-residing, ethnic minority students in middle childhood. Exposure to chronic poverty-related adversity may exacerbate this process, serving as a statistical moderator of the link between NA and children’s internalizing problems.

A third key component of ER is the extent to which children not only appraise situations as upsetting, distressing or fear-inducing but also appraise their own internal feeling states or mood as more negative [22]. Children vary greatly in their struggles and successes with felt experiences of NA [23]. Recent experimental evidence from studies with adults highlights ways in which emotional states can be internally generated through thoughts or internally evoked images, and that those emotional states are accompanied by matching physiological responses (such as changes in eye blink startle) that support the veridicality of subjective report of NA [24]. Negative mood is a hallmark of clinical diagnoses of anxiety and depression among adults and adolescents; recent analyses of children’s NA in middle childhood suggest that linkages between children’s subjective NA and parent-reported internalizing problem may be more complex, with considerable discrepancies between their reports [25]. This study provides us with the opportunity to test the predictive role of children’s NA for their internalizing problems, even after controlling for their accuracy in identifying negative emotions with a large community sample of children.

Multiple theories of emotion highlight the coherent neurobiological organization and coordination of these complex processes as fundamental to the framework of “regulation” [26]. Based on this theoretical framework, children’s behavioral performance on these three components of ER (i.e., ABT, FEA and NA) would be expected to be at least moderately correlated. To date, however, few studies of emotion regulation consider all three of these multiple components, working additively in predicting children’s internalizing problems. Even fewer studies test whether and how these basic models of ER are generalizable to low-income, ethnic minority children at high risk for elevated levels of internalizing problems. This paper attempts to remedy those empirical gaps, carrying out analyses on the ways these ER constructs serve as independent, additive predictors of internalizing problems among the low-income children in our sample in the 5th grade. In addition, we consider whether children’s chronic exposure to poverty-related adversity from early to middle childhood serves to moderate or exacerbate the role of each of those ER processes for their risk of internalizing problems at that 5th grade assessment.

Previous studies have yielded clear evidence that higher exposure to adversities related to living in poverty is associated with alterations in parasympathetic (adrenocortical), emotional, and behavioral response among stressor-exposed children [27,28]. Analyses of the National Longitudinal Survey of Youth, for example, demonstrate that children exposed to chronic poverty are at significantly greater risk of internalizing behavioral problems over time [29]. Although disparate research studies have established links between exposure to chronic poverty, children’s ER skills, and their risk of higher internalizing behavioral difficulties, these studies have not yet been extensively linked or integrated. In the following study, we examine ways that three dimensions of ER and chronic poverty-related adversity may work alone and in combination to predict children’s internalizing problems.

For all sets of analyses, it is important to rule out the possibility of other key hypothesized sources of influence in our models. For example, prior research has also highlighted the role of temperament in contributing to individual differences in children’s ER skills and risk of internalizing behavior problems [30]. For this reason, we include measurement of symptoms of sadness and anxiety in the preschool period (as indicated by parental reports of internalizing symptoms when children were 2–5 years of age) as a rough proxy for temperamental disposition towards negative reactivity. We also control for a host of additional individual, family, and sociocultural factors that might serve as proxies for unobserved (or “omitted”) variables, including child age, child gender, child‘s racial/ethnic identity as African American versus Latina/o, and parent age at baseline.

In sum, we test a number of hypotheses in the following study. First, we hypothesize that children’s emotional regulatory processes involving attention bias to threat, appraisal of facial emotion, and negative affectivity would be moderately intercorrelated. Second, we hypothesized that individual differences in children’s internalizing problems at that 6-year-follow-up assessment would be significantly predicted by those emotional regulatory processes. Third, we hypothesized that the chronicity of children’s exposure to poverty-related adversity from early to middle childhood would serve as an additional statistically significant predictor (and might also moderate, or exacerbate the role of ER difficulties) in predicting children’s internalizing problems in middle childhood. We sought to test those hypotheses using statistically conservative models that would take into account early childhood environmental and constitutional factors that might serve as potential confounds.

## 2. Materials and Methods 

### 2.1. Participants

Six hundred and two children and their families (the majority of whom were African American/Black or Latino/a) were initially enrolled through publicly funded preschools in 7 neighborhoods in the city of Chicago as part of the Chicago School Readiness Project [31]. Of the 602 families originally recruited into the study at baseline, 491 completed the family interview during the 5th grade wave (6 years after baseline). To be included in analyses, children had to have valid data on all of the variables of interest (i.e., parent reported internalizing behavior problems at 5th grade, all measures of ER). Using these criteria, the final analytic sample included a total of 338 children. Preliminary analyses suggest that African American children were slightly more likely to complete the follow-up assessment (representing 70% of the final analytic sample as compared to 66% of the original sample) than were their Latino/a counterparts, *X*^2^ = 6.44, *p* < 0.05. However, the final analytic sample (*N* = 338) did not statistically differ from the original sample across a large number of other family demographic characteristics including family size, maternal age and marital/partnership status, total family income, assets, use of public assistance, and child gender, F(7, 594) = 0.77, *p* = 0.61. 

### 2.2. Procedure

Families were initially recruited through a classroom-based preschool intervention delivered in federally-funded Head Start programs in Chicago (see [31]). The intervention successfully led to the improvement of supportive teacher practices, with children in the treatment group exposed to a significantly higher quality classroom climate, on average, than were children in control group preschool classrooms. Graduate-level research staff obtained families’ written consent for their own and their children’s participation after explaining the nature of the research study, following procedures reviewed and approved by the University of Chicago’s Institutional Review Board for the Protection of Human Subjects. Child- and family-level baseline data in the fall of the preschool year were individually collected through a series of questionnaires from parents by those same research assistants. Preliminary analyses suggest that no statistically significant differences were found between treatment and control group means on any of the baseline or 6-year-follow-up variables included in the present study; thus, children in both treatment and control groups are combined in the following analyses. Demographic characteristics of the analytic sample (including children’s gender and racial/ethnic identity) are provided in Table 1. 

At the 5th grade wave of data collection, parents completed a series of questionnaires, including demographic information and the Behavior Problems Index (BPI; [32]). At that same 5th grade wave of data collection, children participated individually in a 35-min battery of direct assessments adapted from laboratory-based neuroscientific research on ER; this assessment was adapted for field administration on laptop computers in school settings (e.g., lunch room, empty classroom) that could be administered by trained research assistants during the regular school day. Assessments were administered in the following order: Florida Affect Battery (FAB) emotion appraisal tasks to yield FEA scores [33], a dot probe task assessing attentional bias to threatening and positive stimuli to yield ABT scores [34], and the State-Trait Anxiety Inventory for Children (STAIC) anxiety scale to yield NEA scores [35]. Of the 338 children in the final analytic sample, an acceptable level of missingness (ranging from 5%–13%) was found across all predictors and covariates collected during the prior waves, with missing data imputation used for all subsequent analyses [36].

### 2.3. Measures

*Child internalizing problems* were assessed by parents using a shortened version of a well-validated standardized measure, The Behavior Problems Index (BPI) [32]. The original measure consists of 32 items drawn from longer standardized measures (such as the Child Behavior Check-List) that have demonstrated strong psychometric properties of cross-informant and cross-cultural reliability and validity. The current study uses only the parent-reported internalizing subscale, which consisted of 10 items in the baseline year (α = 0.72) and 5 items in the 5th grade year (α = 0.62). Sample items include: “(S/he) is unhappy, sad, or depressed” on a 0–2 scale where 0 = not true, 1 = sometimes true, and 2 = very/often true. Preliminary analysis of parent-reported internalizing subscale at the item and aggregate levels suggest that parents reported low mean levels of symptoms at the 5th grade follow-up (M = 0.21, SD = 0.28), on average, with sufficient variance to support our planned analyses, below (see Table A1).

*Attention bias to threat (ABT).* The Dot Probe task was utilized to measure children’s attention to emotionally negative stimuli [34]. The task consists of a total of 72 test trials divided into 4 blocks. Trials are presented in a semi-random order that is the same across all participants. A trial begins with a fixation cross presented in the center of the screen for 500 ms. Two pictures are then simultaneously presented for 250 ms on the left- and right-hand sides of a computer screen. This short presentation time is intended to capture involuntary, automatic orienting responses to the stimuli. Picture pairs included images drawn from the International Affective Picture database as well as similar images downloaded from the internet and rated for salience by members of the lab [37]. Both positively and negatively valenced images were used, with the present analyses limited to neutral and negatively valenced (i.e., threatening) images only. Threatening images included photographs of threatening animals (e.g., lion, snake, bear, ferocious dog), threatening objects (e.g., handgun), dangerous situations (e.g., house fire, tornado, car crash), and adults displaying threat-related expressions (e.g., woman screaming); each pair of photographs also included at least one neutral image (e.g., table, book). Following the pictures’ presentation, a dot either appears in the same location as the emotional image (congruent trials), in the location opposite the emotional image (incongruent trials), or on either side of the neutral/neutral display (neutral control). The dot remains on the screen for 5000ms or until the participant responds. Respondents use one of two buttons on the keyboard to indicate the location of the dot. Each type of trial type was balanced in terms of presentation on the left vs. right side of the screen. 

For the present analyses, we used data from the threatening and neutral trials only to calculate aggregate scores of attention bias to threat (ABT) [34,38]. Bias was calculated using Equation (1), by subtracting the mean latency to respond to congruent trials from the mean latency to respond to incongruent trials:

Negative bias = ((NGL-GNL) + (GNR-NGR))/2
(1)

where N/G refers to the emotional valence of the image (N = neutral, G = negative/threatening) and the ordering of the letters represents the position of the images on the left side of the screen (the first letter) or the right side of the screen (the second letter). The L/R refers to the position of the probe (L = Left, R = Right). For example, NGL refers to negative image incongruent trials because the probe is presented in the location opposite the emotion image. If participants attend to the emotionally threatening stimulus (G), latencies will be shorter for congruent displays compared to incongruent displays and bias scores will be positive. Conversely, if participants avoid the emotionally negative, threatening image, latencies will be shorter for incongruent displays compared to congruent displays and bias scores will be negative. In our sample, children’s overall ABT scores were negative and were = −11.82 (SD = 51.71) suggesting modest attentional avoidance to threat, on average. 

*Accuracy in facial emotion appraisal (FEA).* Accuracy in appraising emotions from facial expressions was measured using the Facial Affect Battery [33]. FAB stimuli consist of a set of black and white photographs of actors depicting five target emotions (happy, sad, angry, frightened, and neutral). Of all of the photographed faces, only the females met 80% inter-rater reliability criterion regarding the communicative intent of each and every facial expression made by each individual. For this reason the stimuli contained in the FAB are women (rather than men). The stimuli are presented in vertical arrays to minimize potential effects of hemispatial neglect or visuo-attentional scanning disturbance among brain impaired subjects. Twenty trials are given in each of the face emotion tasks. Facial affect selection (Subtest A) assesses the ability to select target facial expressions named by the examiner. On each trial, participants are shown five pictures of different women, expressing each of the five facial emotions. The children are asked identify the picture of the face that corresponds to the emotion named by the examiner (e.g., “point to the angry face”), and the emotion expression which they identify is recorded (whether correct or incorrect). In facial affect discrimination (Subtest B), children are asked, “Are these faces similar or different?” Trial types were randomly scrambled and then presented in fixed order for all participants. The broad, aggregate indicator of accuracy in facial emotion appraisal (FEA) used in these analyses is the percentage of the negative emotion images that children identified correctly across both the FAB A and FAB B subtests. Children’s performance was generally high across both measures, % correct for negative emotion on Subtest A = 0.86 (SD = 0.14), and % correct for negative emotion on Subtest B = 0.89 (SD = 0.14).

*Reports of negative affect (NA)*. Children’s subjective reports of negative affect were measured using the State-Trait Anxiety Inventory for Children [35]. The STAIC consists of two 20-item scales that measure state and trait anxiety in children between the ages of 8 and 14. The Trait scale measures longer-term trait anxiety that has to do with how the child generally feels by prompting children to rate 20 statements such as, “I am secretly afraid” from hardly ever true (1) to often true (3; α = 0.86). The State scale examines short-term state anxiety that is commonly specific to situations. It prompts the child to complete 20 “I feel…” statements on a scale of 1 to 3 with response options such as: 1 = not worried, 2 = worried, 3 = very worried (α = 0.77). A global measure of self-reported negative affect was created by taking average of the two scales. The reliability and validity of these scales has been demonstrated in a sample of urban African-American 3rd and 4th grade children [39]. On average, the children in our sample reported relatively low levels of negative affect on this scale, M = 32.64 (SD = 5.10).

*Poverty-related adversity.* At each of the five waves from preschool through the 6-year-follow-up assessment, parents completed extensive survey questions on eight items, including (a) family household income; (b) family size, and exposure to a range of stressors such as (c) not being able to pay bills; (d)not having heat; (e) not having phone; or (f) electricity shut off; and (g) and (h), two items indicating having not enough money at the end of the month to make ends meet. Following our own and others’ prior research, a cumulative “poverty-related adversity” aggregate was calculated for each wave, summed across waves, and then centered to estimate children’s chronic exposure [40].

*Covariates.* During the baseline (fall of preschool) survey caregivers reported their own age (in years), their child’s, race/ethnicity (1 = African American, 0 = Latina/o), age (in months), and gender (1 = Male, 0 = Female). As mentioned in more detail above, parents also reported their child’s internalizing symptoms during the baseline survey in the preschool year, and we included this as a covariate in models (as described above).

### 2.4. Statistical Analyses

All analyses for this paper were conducted in Stata version 12.1. Bivariate correlations were conducted to examine bivariate associations between ABT, FEA and NA to address our first hypothesis. To address our second and third hypotheses, multivariate Ordinary Least Squares (OLS) regression models were then used to examine whether ABT, FEA and NA, as a set, were significantly and uniquely predictive of children’s internalizing symptoms when they were 8 to 11 years old. Importantly, our model tests whether or not each sub-domain of child ER (i.e., ABT, FEA or NA) is significantly associated with parent-reported child internalizing behavior problems after controlling for demographic and biobehavioral characteristics of children (i.e., child age, gender, race/ethnicity, internalizing behavior problems at baseline) and their parents (i.e., parent age at baseline). We also include a second model testing whether chronic exposure to adversity serves as a statistical moderator of the hypothesized links between three components of ER and children’s internalizing problems. That is, difficulties with ER are expected to be more strongly predictive of internalizing problems for children facing higher levels of poverty-related adversity, relative to their less chronically stressed counterparts.

## 3. Results

### 3.1. Descriptive Analyses

Prior to testing for predicted associations between children’s ER, chronic exposure to poverty-related adversity and internalizing problems at 5th grade, we completed descriptive analyses (see Table 1). 

### 3.2. Tests of Association among ER Measures

Counter to our expectations, the three dimensions of emotion regulation (ER) examined here (ABT, FEA and NA) were not statistically related.

### 3.3. Predicting Children’s Internalizing Symptoms in Middle Childhood from ER and Poverty-Related Adversity

Multivariate OLS regression analyses were carried out (see Model 1, Table 2) to test our second and third hypotheses. It was clearly demonstrated that each of the three hypothesized components of ER played an independent and statistically significant role in predicting children’s internalizing symptoms, after controlling for the roles of exposure to chronic poverty-related adversity and early internalizing symptomatology at age 4. Children showing more avoidance of threatening stimuli on the dot probe were at higher risk of internalizing behavior problems, *b* = −0.03, *SE* = 0.01, *p* < 0.05. In addition, lower accuracy in FEA and higher NA were both associated with higher internalizing symptoms of sadness and anxiety, (*b* = −0.05, *SE* = 0.02 and *b* = 0.05, *SE* = 0.01, respectively, *p* < 0.05). Finally, the chronicity of children’s exposure to poverty-related adversity from early to middle childhood is predictive of higher levels of internalizing problems of sadness and withdrawal in 5th grade. This finding holds even after controlling for children’s early profiles of negative reactivity and their initial levels of symptomatology at age 4. Additional analyses were conducted (see Model 2, Table 2) to test the role of poverty-related adversity as a moderator: Those analyses did not yield any statistically significant evidence for the moderating role of chronic poverty-related adversity.

## 4. Discussion

Our results suggest that a small but significant proportion of the children in our CSRP sample were reported by their parents to struggle with persistent feelings of sadness, worry, and withdrawal as they transition through middle childhood. Our inclusion of neurocognitively oriented assessments helped to elucidate that children showed wide variability in their ability to orient and focus their attention to neutral versus threatening stimuli, and in their ability to accurately appraise facial information. In addition, the low-income children in our sample reported generally low levels of state- and trait-level anxiety during direct assessments. Surprisingly, children’s performance on these three candidate ER processes were statistically uncorrelated, suggesting that they operate independently. This finding differs from previous research linking high levels of negative affectivity with more biased attention to threat (particularly for children with lower levels of regulatory skill, or effortful control) [41]. We look forward to examining whether this set of relations emerges as children transition from middle to high school and into adolescence.

In this study, we tested whether those three neurocognitively-linked processes of emotion regulation worked additively to predict low-income children’s internalizing symptoms. Consonant with prior research, we found that children’s attentional bias away from threat, their lower accuracy in identifying negative facial emotions, and their higher levels of self-reported negative affectivity served as key predictors of their higher behavioral symptoms of sadness, depression, and worry. Results also suggest that children’s chronic exposure to different types of poverty-related adversity over a 6-year period (such as families’ struggles to pay bills and difficulty making ends meet) served as a robust predictor of their risk of internalizing behavior problems in middle childhood. Importantly, poverty-related adversity positively predicted children’s higher levels of internalizing symptoms of sadness, worry, and withdrawal even after statistically taking into account a wide range of potential confounds, including their earlier temperamental negative reactivity in the preschool period. These findings are in keeping with prior research suggesting that insufficient family income (and the multiple stressors that accompany income poverty) take a harsh toll on children’s mental health [42].

Our use of multiple methods across multiple reporters to examine low-income children’s emotion regulation is a strength of this study. In addition to its methodological strengths, this study has several limitations. For example, our models of emotion regulation and risk of internalizing problems among low-income children would have benefited from inclusion of more fine-grained clinical diagnostic measures as parents’ reports of children’s internalizing symptoms have been found to underestimate the severity of child psychiatric risk [39]. In addition, our behavioral data using neuroscientific, lab-developed protocols are likely to be empirically “noisy”, given that multiple sources of measurement error may have been introduced through our effort to collect those data in field-based settings. In our view, these represent a set of important methodological challenges when investigating basic neurobiologically-anchored processes among children facing high risk of exposure to poverty-related adversity. We are confident that these challenges can be overcome in future research and with greater inclusion of low-income children in clinical neuroscientific studies.

## 5. Conclusions

The results of this study have several important theoretical, clinical and policy implications. First, this study provides an important first step in testing theoretical models of the ways that low-income children’s emotional and behavioral development is canalized or shaped, over time [30]. We look forward to continuing to test models of stability versus change in children’s ER processes as we follow CSRP-enrolled children through adolescence. Second, it suggests that clinical support for children exhibiting elevated symptoms of sadness, anxiety, and withdrawal may best be approached in multi-pronged fashion. For example, recent advances in neuroscientifically informed clinical tools called attention bias modification (ABM) that train children in modifying attention biases towards more positive stimuli and away from more threatening stimuli are promising [43]. The results of this study suggest that ABM should be considered along with other tools that clinicians can use to support children’s mental health. Finally, a policy implication is that there is no time to waste in funding and implementing programs designed to buffer children from high levels of poverty-related adversity in early and middle childhood. It is clear that children’s exposure to those forms of adversity represents a pressing public health priority. Intervention for low-income children is needed and has been found to be effective, when delivered early [44]. Many children in poverty go on to demonstrate profiles of resilience, marked by high levels of social and behavioral health—our policies targeting families and children should endeavor to support those pathways to resilience early and in sustained ways across time.

## Figures and Tables

**Table 1:** Sample descriptive statistics (*n* = 338).

Study Variable	*M* (*SD*) %
Internalizing behavior problems (5th grade)	1.04 (1.38)
Emotion Regulation (5th grade)	
ABT	−11.82 (51.71)
FEA	0.89 (0.12)
NA	32.64 (5.10)
Chronic exposure to poverty-rel. adversity (centered)	−0.01 (0.54)
*Covariates*	
Child age at baseline (months)	49.50 (7.24)
Child gender (boy)	46%
Child race/ethnicity (African-American)	70%
Child baseline internalizing behavior problems	3.15 (3.04)
Parent age at baseline (years)	29.72 (7.64)

**Table 2:** Predicting child internalizing behavior problems from poverty-related adversity and three components of ER (*n* = 338).

Predictor	Model 1	Model 2
	Coeff. (SE)	Coeff. (SE)
Emotion regulation		
ABT	−0.03 (0.01) *	−0.03 (0.01)
FEA	−0.05 (0.02) **	−0.05 (0.02) **
NA	0.05 (0.01) ***	0.05 (0.01) ***
Chronic poverty-related adversity	0.09 (0.03) **	0.10 (0.03) **
Interactions		
ABT X Chronic adversity	---	−0.05 (0.03)
FEA X Chronic adversity	---	−0.01 (0.03)
NA X Chronic adversity	---	0.02 (0.11)
Covariates		
Child age baseline (months)	−0.00 (0.00)	−0.00 (0.00)
Child gender (boy)	−0.03 (0.03)	−0.03 (0.03)
Child race/ethnicity		
African American	−0.10 (0.03) **	−0.10 (0.03) **
Parent age baseline (years)	0.00 (0.00)	0.00 (0.00)
Child baseline internalizing behavior	0.04 (0.01) **	0.04 (0.01) **
Baseline income-to-needs ratio (reversed)	−0.06 (0.03)	−0.06 (0.03)
*Constant*	0.47 (0.10) ***	0.46 (0.10) ***

Note. OLS regression coefficients and standard errors presented above, * *p* < 0.05; ** *p* < 0.01; *** *p* < 0.001.

## Appendix A

**Table A1:** Descriptive statistics of parent reported child internalizing behavior problem in middle childhood (*n* = 338) ^1,2^.

Item	“Not True” 0		“Sometimes True” 1		“Very/Often True” 2	
	%	*n*	%	*n*	%	*n*
***5th Grade Internalizing Problems***						
1. Is too fearful or anxious.	64%	217	31%	106	4%	15
2. Feels worthless or inferior.	87%	294	12%	39	1%	5
3. Is unhappy, sad, or depressed.	85%	288	14%	48	1%	2
4. Is withdrawn, does not get involved with others.	89%	302	10%	34	1%	2
5. Cries too much.	81%	275	15%	49	4%	14

^1^ Of sample with valid information on parent reported BPI and direct assessments of students ER; ^2^ Contains missing data (about 13% are missing across items), proportions reflect valid data.
